# Miscellany

**Published:** 1894-12

**Authors:** 


### Miscellany.

---

Civil Service Examinations for Physicians in the State Hospitals.—An open competitive examination of applicants for the positions of first assistant and junior assistant physicians in the state hospital service will be held at the rooms of the State Civil Service Commission, Albany, on Thursday, December 20, 1894, commencing at 10 o'clock, a. m.

Applicants for the position of first assistant physician must be not less than twenty-five years of age, and must have had at least three years' experience in a hospital for the insane. Applicants for the position of junior assistant physician must be not less than twenty-one years of age and have had at least one year's actual experience on the medical staff of a public general hospital or have served at least one continuous year as medical interne in a state hospital for the insane. All applicants must be residents of the State of New York for the year last past and graduates of a legally incorporated medical college.

For application blanks, address the New York Civil Service Commission, Albany, N. Y.

THOMAS CARMODY, *Chief Examiner.*

ALBANY, N. Y., November 22, 1894.

---

VISITING LIST.—Lindsay and Blakiston's Physicians' Visiting List for 1895 has made its appearance. This is the forty-fourth year of its publication, which fact sufficiently tests its popularity. It is published in four forms; one is called the regular edition, arranged for from twenty-five to 100 patients, according to size, and ranging from $1 to $3 in price. Besides this, an interleaved edition, a perpetual edition and a monthly edition are published, covering the various wants of physicians. There is no doubt that for completeness, compactness and simplicity of arrangement it is exceled by none in the market. It is published by P. Blakiston, Son & Co., 1012 Walnut street, Philadelphia, and is sold by all booksellers and druggists.

---

THE publishers of *The Youth's Companion* are offering special prizes for stories by physicians, as follows:

Six prizes will be given for the best stories founded on incidents in a physician's life, as follows: For the best story based upon experiences in a physician's life, $100; for the second, similar story, in merit, $100; for the third in merit, $100; for the fourth in merit, $50; for the fifth in merit, $50; for the sixth in merit, $50. The offers of "special prizes" to physicians include also a very much larger offer of prizes for short original stories made to authors generally. For this reason, a story that secures a special prize in the offer to physicians, if superior to stories offered in the more general competition, will be entitled to both prizes.

Physicians wishing for particulars respecting the character of these stories and the conditions under which prizes will be given should apply by letter, being sure to put M. D. after their names, to Perry, Mason & Co., publishers *Youth's Companion*, Boston, Mass.

---

THE *Medical Mirror* for October devotes considerable space to the publication of an abstract of the proceedings, in part, of the American Association of Obstetricians and Gynecologists at its Toronto meeting, September 19, 20 and 21, 1894. It furnishes very interesting reading and we presume the abstract will be continued in a future issue of this admirable magazine.

---

ALVARENGA PRIZE OF THE COLLEGE OF PHYSICIANS OF PHILADELPHIA.—The College of Physicians of Philadelphia announces that the next award of the Alvarenga prize, being the income for one year of the bequest of the late Señor Alvarenga and amounting to about $180, will be made on July 14, 1895, provided that an essay deemed by the committee of award to be worthy of the prize shall have been offered.

Essays intended for competition may be upon any subject in medicine, but cannot have been published and must be received by the secretary of the college on or before May 1, 1895.

Each essay must be sent without signature, but must be plainly marked with a motto and be accompanied by a sealed envelope having on its outside the motto of the paper and within it the name and address of the author.

It is a condition of competition that the successful essay or a copy of it shall remain in possession of the college; other essays will be returned upon application within three months after the award.

The Alvarenga prize for 1894 has been awarded to Dr. G. E. de Schweinitz, of Philadelphia, for his essay entitled Toxic Amblyopias. CHARLES W. DULLES, *Secretary*.

